# Default mode network in young male adults with autism spectrum disorder: relationship with autism spectrum traits

**DOI:** 10.1186/2040-2392-5-35

**Published:** 2014-06-11

**Authors:** Minyoung Jung, Hirotaka Kosaka, Daisuke N Saito, Makoto Ishitobi, Tomoyo Morita, Keisuke Inohara, Mizuki Asano, Sumiyoshi Arai, Toshio Munesue, Akemi Tomoda, Yuji Wada, Norihiro Sadato, Hidehiko Okazawa, Tetsuya Iidaka

**Affiliations:** 1 Developmental Emotional Intelligence, Division of Developmental Higher Brain Functions, Department of Child Development United Graduate School of Child Development, Osaka University, Kanazawa University, Hamamatsu University School of Medicine, Chiba University and University of Fukui, Fukui, Eiheiji 910-1193, Japan; 2 Research Center for Child Mental Development, University of Fukui, Fukui, Eiheiji 910-1193, Japan; 3 Department of Neuropsychiatry, Faculty of Medical Sciences, University of Fukui, Fukui, Eiheiji 910-1193, Japan; 4 Biomedical Imaging Research Center, University of Fukui, Fukui, Eiheiji 910-1193, Japan; 5 Department of Child and Adolescent Mental Health, National Institute of Mental Health, National Center of Neurology and Psychiatry, Kodaira, Tokyo 187-8553, Japan; 6 Graduate School of Engineering, Osaka University, Suita, Osaka 565-0871, Japan; 7 Research Center for Child Mental Development, Kanazawa University, Kanazawa, Ishikawa 920-8641, Japan; 8 Department of Cerebral Research, National Institute for Physiological Sciences, Okazaki, Aichi 444-8585, Japan; 9 Department of Psychiatry, Graduate School of Medicine, Nagoya University, Nagoya, Aichi 466-8550, Japan

**Keywords:** Autism spectrum disorder (ASD), Autism spectrum traits, Autism-spectrum quotient (AQ), Default mode network (DMN), Resting-state functional connectivities (rs-FCs), Anterior medial prefrontal cortex (aMPFC), Posterior cingulate cortex (PCC)

## Abstract

**Background:**

Autism spectrum traits are postulated to lie on a continuum that extends between individuals with autism and individuals with typical development (TD). Social cognition properties that are deeply associated with autism spectrum traits have been linked to functional connectivity between regions within the brain’s default mode network (DMN). Previous studies have shown that the resting-state functional connectivities (rs-FCs) of DMN are low and show negative correlation with the level of autism spectrum traits in individuals with autism spectrum disorder (ASD). However, it is unclear whether individual differences of autism spectrum traits are associated with the strength of rs-FCs of DMN in participants including the general population.

**Methods:**

Using the seed-based approach, we investigated the rs-FCs of DMN, particularly including the following two core regions of DMN: the anterior medial prefrontal cortex (aMPFC) and posterior cingulate cortex (PCC) in 19 young male adults with high-functioning ASD (mean age = 25.3 ± 6.9 years; autism-spectrum quotient (AQ) = 33.4 ± 4.2; full scale IQ (F-IQ) = 109.7 ± 12.4) compared with 21 age- and IQ-matched young male adults from the TD group (mean age = 24.8 ± 4.3 years; AQ = 18.6 ± 5.7; F-IQ = 109.5 ± 8.7). We also analyzed the correlation between the strength of rs-FCs and autism spectrum traits measured using AQ score.

**Results:**

The strengths of rs-FCs from core regions of DMN were significantly lower in ASD participants than TD participants. Under multiple regression analysis, the strengths of rs-FCs in brain areas from aMPFC seed showed negative correlation with AQ scores in ASD participants and TD participants.

**Conclusions:**

Our findings suggest that the strength of rs-FCs in DMN is associated with autism spectrum traits in the TD population as well as patients with ASD, supporting the continuum view. The rs-FCs of DMN may be useful biomarkers for the objective identification of autism spectrum traits, regardless of ASD diagnosis.

## Background

Autism spectrum disorder (ASD) is a complex neurodevelopmental disorder characterized by impaired social communication and social interaction, and unusually restricted, repetitive behaviors and interests [1,2]. These characteristics are postulated to lie on a continuum that extends between individuals with autism and individuals with typical development (TD) [3,4]. This continuum view suggests the possibility that autism spectrum traits are found not only at high levels in individuals with ASD but also at lower levels among individuals without ASD [5-7]. This continuum view shifts us away from merely categorical diagnosis towards the quantitative support of daily difficulties associated with autism spectrum traits of each individual regardless of ASD diagnosis. Taking the quantitative support into consideration necessitates the use of an instrument that can quantify autism spectrum traits in the entire population; such an instrument could also be used to define the broader autism phenotype [8,9].

To identify the level of autism spectrum traits, several instruments have been developed. The autism-spectrum quotient (AQ) questionnaire is a useful instrument that was developed for identifying the extent of autistic spectrum traits, assuming that adults of normal intelligence may have autism spectrum traits at various levels [10]. Moreover, AQ is a validated measure of autism spectrum characteristics found within both the typical population and individuals with a diagnosis of autism [10,11].

Recent behavioral studies have shown that individual differences in autism spectrum traits measured using AQ are associated with the performance of social cognition processing tasks such as self-focused attention, mind reading, episodic memory, and inferring others’ mental state in both a population with ASD and that with TD [12-15]. Neuroimaging studies also demonstrate that autism spectrum traits measured using AQ are associated with structural and functional abnormalities in brain regions including the insula, inferior frontal gyrus, and posterior cingulate cortex (PCC), which are involved in social cognition processing in individuals with and without ASD [16-18]. Taken together, these findings indicate that it is very important to evaluate the neuroimaging studies considering the level of autism spectrum traits.

Social and self-referential cognitive processes have been linked with cortical midline brain regions such as PCC and the medial prefrontal cortex (MPFC), reflecting the high functional connectivities within the default mode network (DMN) [19,20]. In healthy individuals with TD, the functional connectivities of DMN are consistently deactivated when the individual is engaged in a cognitively demanding, goal-oriented task [21]. On the other hand, brain regions of the DMN are often engaged during social cognition processing tasks such as self-reference and the ‘theory of mind’ [22-24]. This marked overlap between brain regions of the DMN and regions of the ‘social brain’ suggests that the brain regions of the DMN are strongly associated with the social cognition process [25,26].

Several studies of the DMN in individuals with ASD have shown its lower functional connectivities in resting-state functional magnetic resonance imaging (rs-fMRI), using the approaches of both region of interest (ROI) analysis and independent component analysis (ICA), than in individuals with TD [27-32]. In addition, these studies also showed negative correlations between the strength of the resting-state functional connectivity (rs-FC) of DMN and autism spectrum traits including social deficits in individuals with ASD. However, these studies have primarily investigated correlations between the strength of rs-FCs of DMN and autism spectrum traits only in individuals with ASD [27,29,31]. To the best of our knowledge, the individual differences in autism spectrum traits regarding social cognition processing, which may also be associated with the strength of rs-FCs of DMN in individuals with or without ASD, have not been evaluated.

The aims of this study were: (1) to clarify the rs-FCs of DMN in high-functioning young male adults with ASD by comparison with age- and IQ-matched young male adults with TD on the basis of the location of ROIs within DMN during rs-fMRI; and (2) to evaluate correlations between the strength of rs-FCs of DMN and autism spectrum traits measured using AQ scores in TD participants and ASD participants, respectively.

## Methods

### Participants

Nineteen male individuals with high-functioning ASD were recruited by the Department of Neuropsychiatry at the University of Fukui Hospital, Japan, and the Department of Psychiatry and Neurobiology of the Kanazawa University Hospital, Japan. The authors (HK and TMu) diagnosed the participants on the basis of the criteria in the Diagnostic and Statistical Manual of Mental Disorders (DSM-IV-TR) [33] and standardized criteria taken from the Diagnostic Interview for Social and Communication Disorders (DISCO) [34]. The DISCO is reported to have good psychometric properties [35]. It also contains items on early development, and a section on activities of daily life, thereby giving the interviewer an idea of the individual’s level of functioning in several different areas, not only social functioning and communication [35]. All participants were right-handed [36]. Twenty-one age-matched and intelligence quotient (IQ)-matched TD male participants were recruited from the local community. Individuals with a history of major medical or neurological illness, including epilepsy, significant head trauma, or a lifetime history of alcohol or drug dependence, were excluded from this study. They were screened to exclude individuals who had a first degree relative with an Axis I disorder, diagnosed on the basis of the DSM-IV criteria. IQ was assessed using the Wechsler Adult Intelligence Scale-III (WAIS-III) [37]. All the participants had full scale IQ (F-IQ) scores >85. There were no significant differences between the groups in terms of age, verbal IQ, performance IQ, and F-IQ (all *P* >0.5). All the participants also completed the AQ questionnaire [10]. The protocol used for this study was approved by the ethics committee of the University of Fukui. After a complete explanation of the study, all the participants provided written, informed consent. Their mean age, handedness, IQ and AQ score are shown in Table 1.

**Table 1:** **Demographic data**, **IQ and AQ scores of participants**

**Measure**	**ASD participants**	**TD participants**
	**(n = 19)**	**(n = 21)**
Age (SD)	25.3 (6.9)	24.8 (4.3)
Age (range)	(16-40)	(19-35)
Handedness^a^ : Right / Left	19/0	21/0
IQ^b^		
F-IQ (SD)	109.7 (12.4)	109.5 (8.7)
V-IQ (SD)	113.2 (15.7)	111.2 (10.6)
P-IQ (SD)	103.9 (12.2)	105.3 (9.7)
Autism spectrum traits		
Total AQ^c^ (SD)	33.4 (4.2)	18.6 (5.7)
Social skills^c^	8.3 (1.5)	3.5 (2.3)
Communication^c^	7.2 (1.5)	2.7 (2.1)
Attention switching^c^	7.4 (1.8)	4.8 (1.5)
Imagination^c^	5.8 (2.2)	3.9 (1.5)
Attention to detail	4.7 (2.2)	3.7 (1.9)

^a^Assessed by the Edinburgh handedness inventory [36]. All participants were right-handed.

^b^Intelligence quotient was measured using the Wechsler Adult Intelligence Scale-Third Edition in all the participants.

^c^*P* <0.01, with the independent-samples t-test, comparison between participants with ASD and TD.

AQ, autism spectrum quotient; ASD, autism spectrum disorder; F*-*IQ, full scale IQ; P*-*IQ, performance IQ; TD, typical development; V*-*IQ, verbal IQ.

### fMRI data acquisition

Functional images were acquired with T2*-weighted gradient-echo echo-planar imaging (EPI) sequence using a 3-T imager (Discovery MR 750; General Electric Medical Systems, Milwaukee, WI, USA) and a 32-channnel head coil. Two hundred and one volumes were acquired in each participant. Each volume consisted of 40 slices, with a thickness of 3.5 mm and a 0.5 mm gap to cover the entire brain. The time interval between two successive acquisitions of the same slice (repetition time, TR) was 2,300 ms, with an echo time (TE) of 30 ms and a flip angle (FA) of 81 degrees. The field of view (FOV) was 192 × 192 mm and the matrix size was 64 × 64, giving volume dimensions of 3 × 3 mm. The participants were instructed to close their eyes but stay awake and think of nothing in particular. A total of 201 volumes were acquired for a total imaging time of 7 min 42 s. The experiment was conducted at the Biomedical Imaging Research Center of the University of Fukui.

### fMRI data analysis

#### Preprocessing

Data were analyzed using SPM8 software (Wellcome Department of Imaging Neuroscience, London, UK). After discarding the first five volumes, all volumes were realigned spatially to the mean volume, and the signal from each slice was realigned temporally to that obtained from the middle slice using sinc interpolation. The resliced volumes were normalized to the Montreal Neurological Institute (MNI) space with a voxel size of 2 × 2 × 2 mm using the EPI template of SPM8. The normalized images were spatially smoothed with a 6-mm Gaussian kernel.

Rs-fMRI datasets were processed using a toolkit of the Data Processing Assistant for Resting-State fMRI (DPARSF; http://www.restfmri.net) [38]. We conducted additional processing as follows: (1) removing the linear trend in the time series; and (2) performing temporally bandpass filtering (0.01-0.08 Hz) to reduce the effects of low-frequency drift and high-frequency noise [39,40]. To control the non-neural noise in the time series [41]; (3) several sources of spurious variance, that is, six parameters from the rigid body correction of head motion, white matter signals, CSF signals, and global signals were removed from the data through linear regression [42].

#### Head movement parameters

Rs-FCs of DMN are significantly affected by the head motion of participants during fMRI scanning; that is, long-distance correlations are decreased by participant motion, whereas many short-distance correlations are increased [43-47]. To investigate the effect of head motion and motion artifacts in rs-FCs, the root mean square (RMS) of six movement parameters obtained in the realignment process (x-, y-, z translations and x-, y-, z rotations), mean frame-to-frame RMS motion [43] and frame-wise displacement (FD) [45] were calculated for each participant. There were no significant differences in RMS (*P* values ranged from 0.17 to 0.70), mean frame-to-frame RMS motion (*P* value was 0.11) and FD (*P* value was 0.14) between the groups. In addition, there is no significant relationship between AQ scores and the six RMS head movement parameters (*P* values ranged from 0.10 to 0.78).

#### Definition of ROIs

To clarify the rs-FCs of DMN in the present study, we defined the regions in the anterior MPFC (aMPFC) and PCC as ROIs. The ROI coordinates were selected from the DMN meta-analysis [48]. The seed regions of the aMPFC and PCC comprise core seeds within the functional connectivity of DMN, and their widespread connectivities are supported by connectional anatomy studies [48-50]. Two spherical ROIs of 8 mm radius centered at a coordinate listed in Table 2 were drawn for each participant in line.

**Table 2:** **Significant differences in rs**-**FCs between groups with ASD and TD** (**TD** >**ASD**)

**Seed region**	**Region**	**MNI coordinates**			**Z**-**score**	**Cluster size**
		**x**	**y**	**z**		***kE* (voxels)**
aMPFC		-6	52	-2		
	Paracentral lobule	4	-30	72	4.21	138
	Paracentral lobule	-10	-36	74	4.11	176
	Middle frontal gyrus	-24	25	48	4.51	89
PCC		-8	-56	26		
	Medial prefrontal cortex	-6	54	-12	3.92	82

The statistical threshold for contrasts was *P* <0.001 uncorrected for height and cluster *P* <0.05 corrected for multiple comparisons.

aMPFC, anterior medial prefrontal cortex; MNI, Montreal Neurological Institute; PCC, posterior cingulate cortex.

#### Rs-FC analysis

**Figure 1:**
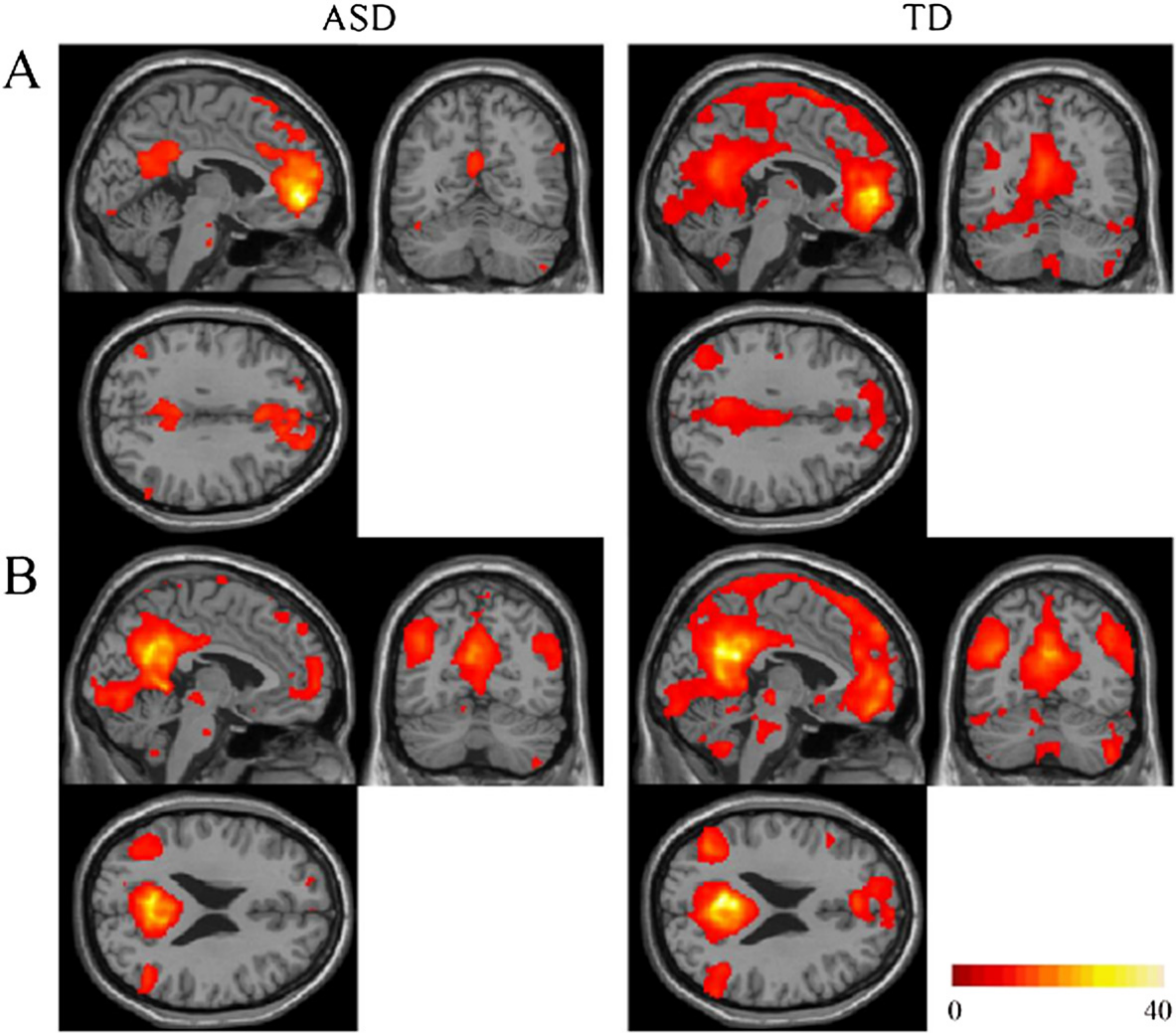
**Results of rs**-**FCs from the aMPFC or PCC seed for each group. (A)** Rs-FCs from the aMPFC seed ROI are shown for each group. **(B)** Rs-FCs from the PCC seed ROI are shown for each group. The statistical threshold for both results was set at *P* = 0.05 with FWE correction and *k* = 10 voxels for presentation purpose. The region with functional connectivities was smaller in the ASD group than in the TD group. The coordinates **(A)** (-3, -60, 32); **(B)** (-4, -65, 24) at the figure) were selected from a previous study [52]. Color bar denotes t-statistic range.

After the processing of rs-fMRI data, we used the predefined seed regions for voxel-wise rs-FC analyses using the DPARSF toolbox. The mean time course of all voxels in each seed ROI was used to calculate voxel-wise linear correlations (Pearson’s correlation) in the whole brain during the rs-fMRI period. Individuals’ r values were normalized to z values using Fisher’s z transformation. At the group level, each image pertaining to z values reflecting a correlation between the seed ROI and each voxel of the brain was entered into a random effect two-sample t-test to identify the whole-brain regions showing significant differences between the groups with ASD and TD. In seed-based rs-FC analyses, global time series, computed across all brain voxels, along with six motion parameters, were used as additional covariates to remove confounding effects of physiologic noise and participant movement. For the presentation purpose, rs-FCs from the aMPFC and PCC seed ROIs are shown separately for each group at the threshold of *P* = 0.05 with a family wise error correction (Figure 1). Results were corrected between group comparisons, set at *P* = 0.001, uncorrected at peak level, and *P* = 0.05 with cluster level.

#### Relationships among rs-FCs of group differences with AQ or IQ

To confirm the relationship between the significant difference in strength of rs-FCs and autism spectrum traits or IQ, we calculated correlations between AQ scores, F-IQ and the Z values, which were extracted from the regions showing the strength of rs-FCs of significant group differences (Spearman’s rank order correlation coefficients; the statistical threshold was set at *P* = 0.01).

#### Multiple regression analysis among the strength of rs-FC with AQ, IQ, or age

To investigate the relationships among the strength of rs-FC, autism spectrum traits, IQ, or age across each group, we performed additional analyses of nine independent variables in a multiple regression analysis in SPM8, AQ total scores, F-IQ, age, and RMS of six movement parameters as covariates. The statistical threshold for contrasts was *P* <0.001 uncorrected for height and cluster *P* <0.05 corrected for multiple comparisons. In addition, we tested whether the five AQ subscale scores would also show correlations with the brain regions and we observed correlations in each group.

## Results

### Differences in the strength of rs-FCs in DMN between groups

Rs-FCs from the aMPFC and PCC seed regions were distributed in the medial parts of the brain (Figure 1). In the ASD group compared with the TD group, substantially smaller areas were functionally connected with these seed regions.

**Figure 2:**
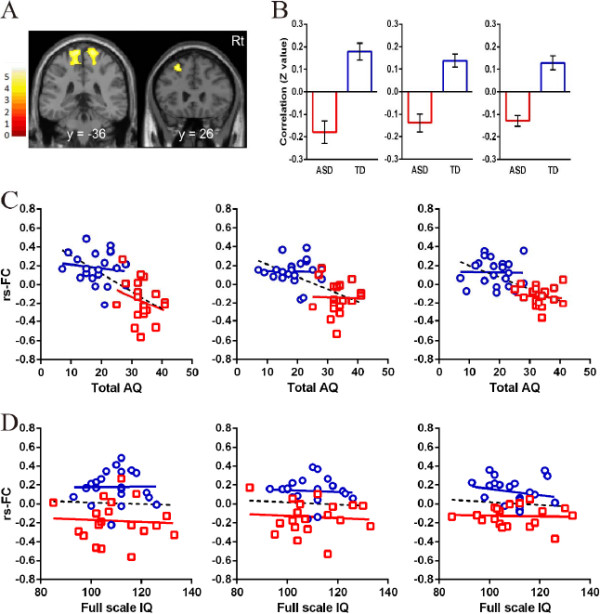
**Results of rs**-**FCs from the aMPFC seed ROI between groups. (A)** Comparison of rs-FCs from the aMPFC seed ROI between groups. There were significant clusters in the paracentral lobuli (left) and middle frontal gyrus (right). Detailed information on these clusters is shown in Table 2. **(B)** Mean and one standard error of Fisher’s z-transformed correlation coefficients extracted from the left paracentral lobule (left), right paracentral lobule (middle), and middle frontal gyrus (right) clusters are shown for each group. Rs-FCs were lower in the ASD group than in the TD group. **(C)** Scatter plots showing the correlations of the strength of rs-FCs in the aMPFC with left paracentral lobule (left), right paracentral lobule (middle), and MFG (right) with AQ total scores in each group. **(D)** Scatter plots showing the correlations of the strength of rs-FC in the aMPFC with left paracentral lobule (left), right paracentral lobule (middle), and MFG (right) with full-scale IQ score in each group. The red bar and squares represent individuals with ASD, and the blue bar and circles represent participants with TD. Color bar denotes t-statistic range.

**Figure 3:**
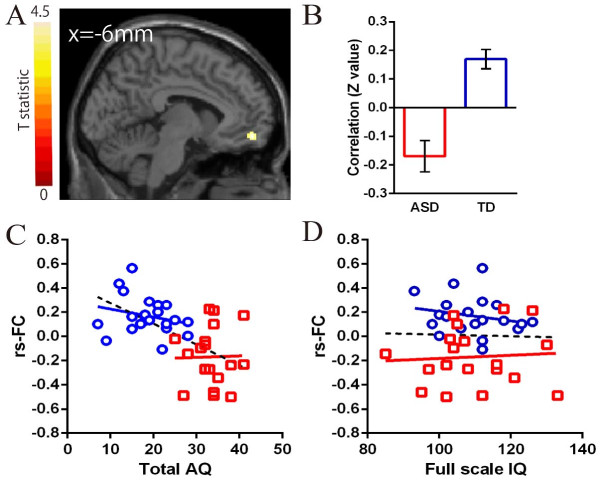
**Results of rs**-**FCs from the PCC seed ROI between groups. (A)** Comparison of rs-FCs from the PCC seed ROI between groups. There was a significant difference in the medial prefrontal cortex. Detailed information on the cluster is shown in Table 2. **(B)** Mean and one standard error of Fisher’s z-transformed correlation coefficients extracted from the medial prefrontal cortex are shown for each group. Rs-FCs were lower in the ASD group than in the TD group. **(C)** Scatter plots showing the correlations of the strength of rs-FC in the PCC with MPFC with AQ total scores in each group. **(D)** Scatter plots showing the correlations of the strength of rs-FCs in the PCC with MPFC with full-scale IQ score in each group. The red bar and squares represent individuals with ASD, and the blue bar and circles represent participants with TD.

In the ROIs within the DMN, significant differences in the strength of rs-FCs from the aMPFC seed were observed in the paracentral lobuli and middle frontal gyrus (MFG) between the two groups (Table 2, Figure 2). A significant difference in rs-FCs from the PCC seed was observed in the MPFC between the groups (Table 2, Figure 3). The mean correlation coefficient (Fisher z-transformed) for each group is shown in Figure 2 and Figure 3. The strengths of all of these rs-FCs were lower in the ASD group compared with the TD group. Individuals with ASD showed no significantly higher strength of rs-FCs from the seed regions compared with the TD group.

### Relationship between the strength of rs-FCs of group differences and AQ or IQ

In the ASD group, there were no significant correlations between either the strength of rs-FCs and F-IQ or AQ total score (Figure 2; Figure 3, *P* >0.01). In the TD group, there were also no significant correlations between either the strength of rs-FCs and F-IQ or AQ total score (Figure 2; Figure 3, *P* >0.01).

### Relationship with AQ or IQ in the multiple regression analysis

**Figure 4:**
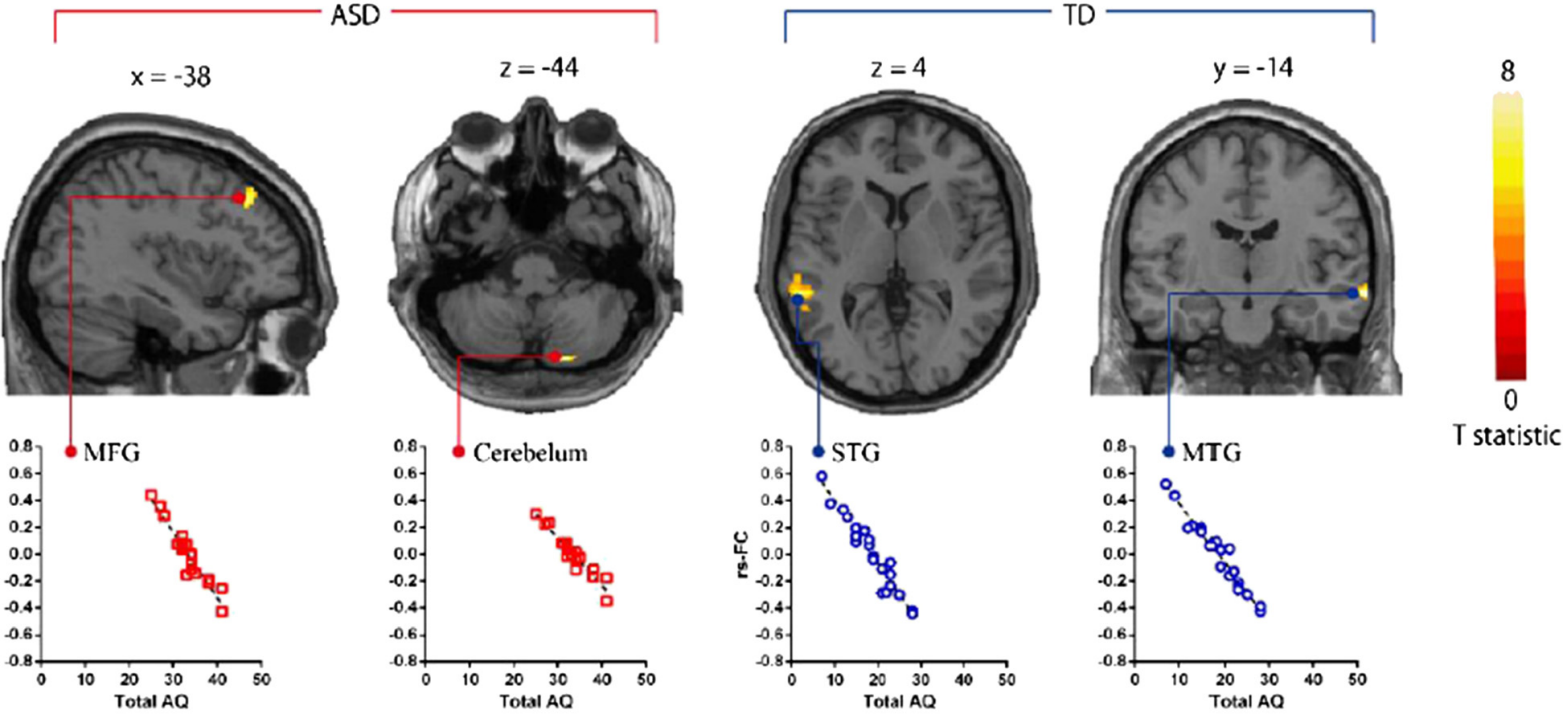
**Brain regions showing negative correlations between AQ and the strength of rs**-**FCs from aMPFC seed in each group.** Brain regions showing negative correlations between AQ and strength of rs-FCs from aMPFC seed in multiple regression analysis. The statistical threshold for contrasts was *P* <0.001 uncorrected for height and cluster *P* <0.05 corrected for multiple comparisons. The scatterplots show the association of AQ and the strength of rs- FCs in selected regions. aMPFC, anterior medial prefrontal cortex; MFG, middle frontal gyrus; MTG, middle temporal gyrus; STG, superior temporal gyrus; rs-FC, resting state functional connectivity.

In the ASD group, AQ total scores were significantly negatively correlated with the strength of rs-FC in aMPFC with MFG and cerebellum (Table 3; Figure 4, *P* <0.01). We also found that two AQ subscale scores (communication scores and attention switching scores) also significantly negatively correlated with the strength of rs-FCs in aMPFC with MFG and cerebellum (*P* <0.05). In the TD group, AQ total scores were significantly negatively correlated with the strength of rs-FC in aMPFC with superior temporal gyrus (STG) and MTG (Table 3; Figure 4, *P* <0.01). We also found that three AQ subscale scores (social skill scores, communication scores, and attention switching scores) also significantly negatively correlated with the strength of rs-FCs in aMPFC with STG and MTG (*P* <0.01).

**Table 3:** **Brain regions showing negative correlations between AQ and the strength of rs**-**FCs in multiple regression analysis**

**Seed region**	**Region**	**MNI coordinates**			**Z**-**score**	**Cluster size**
		**x**	**y**	**z**		***kE* (voxels)**
**ASD participants**						
aMPFC	Middle frontal gyrus	-46	26	46	4.28	101
	Cerebellum	18	-82	-44	4.32	86
PCC	None					
**TD participants**						
aMPFC	Superior temporal gyrus	-58	-40	4	4.12	112
	Middle temporal gyrus	68	-14	-12	4.59	106
PCC	None					

The statistical threshold for contrasts was *P* <0.001 uncorrected for height and cluster *P* <0.05 corrected for multiple comparisons.

aMPFC, anterior medial prefrontal cortex; MNI, Montreal Neurological Institute; PCC, posterior cingulate cortex.

There were no significant correlations between the strength of rs-FCs and each participant’s F-IQ in all the participants, in the ASD group or in the TD group, in the multiple regression analysis (*P* >0.01).

### Relationship with age in the multiple regression analysis

**Figure 5:**
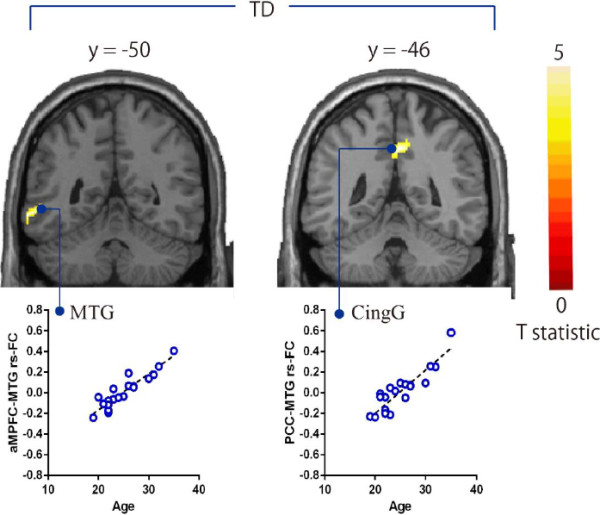
**Brain regions showing positive correlations between age and the strength of rs**-**FCs from seed regions in TD participants.** Brain regions showing positive correlations between age and the strength of rs-FCs from seed regions in multiple regression analysis. The statistical threshold for contrasts was *P* <0.001 uncorrected for height and cluster *P* <0.05 corrected for multiple comparisons. The scatterplots show the association of age and the strength of rs-FCs in selected regions. aMPFC, anterior medial prefrontal cortex; CingG, cingulate gyrus; MTG, middle temporal gyrus; PCC, posterior cingulate cortex; rs-FC, resting state functional connectivity.

In the TD group, age was significantly positively correlated with the strength of rs-FCs in the aMPFC with MTG and PCC with cingulate gyrus (Table 4; Figure 5). We found no significant correlations between age and the strength of rs-FCs of DMN, in the ASD group or all the participants.

**Table 4:** **Brain regions showing correlations between age and the strength of rs**-**FCs in multiple regression analysis**

**Seed region**	**Region**	**MNI coordinates**			**Z-score**	**Cluster size**
		**x**	**y**	**z**		***kE *** **(voxels)**
**All participants (Positive and Negative correlations)**						
NONE						
**ASD participants (Positive and Negative correlations)**						
NONE						
**TD participants (Positive correlation)**						
aMPFC	Middle temporal gyrus	-62	-50	-6	4.26	71
PCC	Cingulate gyrus	2	-46	42	3.91	90
**TD participants (Negative correlation)**						
NONE						

The statistical threshold for contrasts was *P* <0.001 uncorrected for height and cluster *P* <0.05 corrected for multiple comparisons.

aMPFC, anterior medial prefrontal cortex; MNI, Montreal Neurological Institute; PCC, posterior cingulate cortex.

## Discussion

We found that young male adults with high-functioning ASD display lower rs-FCs between two seeds (aMPFC and PCC) of DMN and other brain regions In addition, we found that autism spectrum traits measured using the AQ score are associated with the strength of rs-FCs of DMN that contain brain regions relevant to social cognition processing, in each TD participants and ASD participants, respectively. These results complement previous studies [26,29,31], suggesting that the strengths of rs-FCs of DMN are associated with autistic spectrum traits of social cognition processing in ASD participants. In addition, our results showed that this association between the strength of rs-FCs of DMN and autistic spectrum traits also exists not only in ASD participants but also in TD participants. These findings suggest that the strength of rs-FCs of DMN may underlie some of the autism spectrum traits, regardless of ASD diagnosis.

### Difference in functional connectivities of DMN between ASD and TD groups

Our results mostly agree with those of other studies of rs-FCs of DMN [27-32] that demonstrated lower rs-FCs of DMN in ASD. However, as for MPFC, the regions showing lower functional connectivities in previous studies are not always in agreement with those found in this study [27-30,32]. Differences in approach (seed-based or ICA) might have made direct comparisons between results somewhat challenging [51]. Andrews-Hanna *et al.*[49] demonstrated that the aMPFC of DMN is associated with social cognition processes including judgments or remembering trait adjectives about themselves compared with other people, whereas the dorsal medial prefrontal cortex (dMPFC) of DMN is associated with social cognition processes including self-referential judgments about their present situation or mental states. These findings taken together with our findings suggest that rs-FCs in brain regions including both aMPFC and dMPFC are associated with autism spectrum traits associated with processes of basic social cognition of self and others in individuals with ASD.

### DMN associated with autism spectrum traits

In recent studies of rs-FCs of DMN in ASD, correlations have been investigated between autism spectrum traits and functional connectivities within brain areas such as the precuneus [27], MPFC [27,29,32], anterior cingulate cortex [27], and superior frontal gyrus [31]. However, most of these studies analyzed these correlations in only ASD participants. In general, these studies suggest negative correlation between the strength of rs-FCs of DMN and autism spectrum traits in individuals with ASD.

In the current findings, the strength of rs-FCs in brain areas from aMPFC seed showed negative correlations with AQ total score not only in ASD participants but also in TD participants in the multiple regression analysis, although these areas from aMPFC seed that showed negative correlations with AQ total scores in two groups were different from each other (Table 3). Moreover, among AQ subscale scores, communication scores and attention switching scores were related to the strength of rs-FCs of DMN in the ASD group. On the other hand, social skill scores, communication scores, and attention switching scores were related to the strength of rs-FCs of DMN in the TD group. Our findings suggest that the strength of rs-FCs of DMN might underlie the level of autism spectrum traits in participants without ASD diagnosis or with subthreshold autism spectrum traits, supporting the continuum view. In addition, considering the results of the multiple regression analysis using AQ subscale scores, the nature of autism spectrum traits, which affects the strength of rs-FCs of DMN in aMPFC seed, might be different in each group and the function of DMN might not always be the same between ASD and TD groups.

### Age-related changes in functional connectivities of DMN

Particularly interesting from our viewpoint is the lack of consensus regarding the strength of rs-FC aspects of age variation with ASD. In relation to age-related changes in DMN in ASD, few studies have examined age correlates of functional connectivities by rs-fMRI. To the best of our knowledge, only one rs-fMRI study has examined the functional connectivities in children with ASD compared with age- and IQ-matched children with TD [52]. The previous study showed higher rs-FCs of DMN in PCC seeds in children with ASD than in children with TD [52], but we found that lower rs-FCs of DMN in PCC seeds in the ASD group and a relationship between rs-FCs of DMN in PCC seeds and age in the TD group. We speculate that the age-related changes in the functional connectivities of DMN in ASD and TD may be linked to differences in neurodevelopmental mechanisms in childhood and numerous variables including point of development. Future studies are required to explore the variation of rs-FCs of DMN with age for individuals with ASD.

### Future directions

First, the size of each group in this study may be relatively too small to demonstrate a relationship between the various levels of autism spectrum traits and the strength of rs-FCs of DMN. Second, we defined the seed regions for rs-FC analyses on the basis of the location of ROIs within the DMN in accordance with a previous study. Although the seed-based analysis in the present research makes it possible to determine rs-FC precisely, this analysis did not reveal information about intrinsically connected networks and their interactions [51]. Future study is necessary to replicate these findings, using the ICA approach for exploring information about intrinsically connected networks. Third, we included global signal regression in generating the FC map. However, it is a quite controversial issue whether global signal regression changes resting-state correlations and produces negative correlations [53]. Thus, the findings used in the present study are not definitive, and future study is necessary to compare the results with confound regression strategies. Finally, our participants were only young male adults with high-functioning ASD. Future study is needed to clarify rs-FCs in both males and females, children with ASD, and ASD individuals without high-functioning. These additional researches will help provide more complete pictures that may clarify the etiology of ASD.

## Conclusions

Young male adults with high-functioning ASD showed lower rs-FCs of DMN compared with age- and IQ-matched young male adults with TD. Moreover, the strength of rs-FCs of DMN was associated with autism spectrum traits in each ASD and TD group, regardless of ASD diagnosis. We propose that the strength of rs-FCs of DMN might underlie the level of autism spectrum traits and might be one of the potential biomarkers for the objective identification of the level of autism spectrum traits, regardless of ASD diagnosis.

## Abbreviations

AMPFC: Anterior medial prefrontal cortex; AQ: Autism-spectrum quotient; ASD: Autism spectrum disorder; DMN: Default mode network; DMPFC: Dorsal medial prefrontal cortex; IQ: Intelligence quotient; PCC: Posterior cingulate cortex; Rs-FC: Resting-state functional connectivities; TD: Typical development.

## Competing interests

The authors declare that they have no competing interests.

## Authors’ contributions

MJ was involved in conducting the experiment, analyzing and interpreting data, and drafting the article. HK was involved in recruiting the participants, diagnosing the participants with ASD, conducting the experiment, analyzing and interpreting data, and drafting the article. DNS, TM (fifth author), and KI were involved in recruiting the participants and conducting the experiment. MI was involved in recruiting the participants, interpreting data, and drafting the article. TM (eighth author) was involved in recruiting the participants and diagnosing the participants with ASD. MA, SA, AT, YW, NS, and HO were involved in interpreting the data. TI was involved in designing, analyzing and interpreting data, and drafting the article. All the authors have read and approved the final manuscript.
